# Cardiac tamponade and successful pericardiocentesis in an extremely low birth weight neonate with percutaneously inserted central venous line: a case report

**DOI:** 10.1186/1757-1626-3-15

**Published:** 2010-01-11

**Authors:** Alfredo Pizzuti, Emilia Parodi, Paola Abbondi, Mario Frigerio

**Affiliations:** 1 Cardiology Unit, Ordine Mauriziano Hospital, (Largo Turati n 62), Turin, (10128), Italy; 2 Neonatal Intensive Care Unit, Ordine Mauriziano Hospital, (Largo Turati n 62), Turin, (10128), Italy

## Abstract

**Background:**

Pericardial effusion and cardiac tamponade are rare but life-threatening complications of percutaneosuly inserted central line (PICL) use in extremely low birth weight (ELBW) neonates, with an incidence reported between 0.07% and 2% of PICLs placement. Timely diagnosis and pericardiocentesis has been proven to be life-saving.

**Case presentation:**

The patient was a 620 g birth weight neonate who presented with sudden cardiac instability 18 days after the insertion of a PICL and in spite of a presumed satisfactory position of the catheter tip.

The transthoracic echocardiography demonstrated severe pericardial effusion with evidence of cardiac tamponade.

Successful urgent subxiphoid pericardiocentesis was performed; totally 2 ml of whitish fluid was collected, which resulted consistent to the composition of the hyperosmolar TPN solution infused.

**Conclusion:**

Cardiac tamponade should be considered in any newborn with a peripherally inserted central catheter who presents with cardiorespiratory instability (bradycardia, cyanosis and metabolic acidosis), even when lines are believed to be placed correctly.

## Background

Percutaneously inserted central lines (PICLs) are commonly used in neonatal intensive care units (NICU) to provide parenteral nutrition and medications for extremely low birth weight (ELBW) infants. PICLs have been associated with a number of device-specific complications (occlusion, infection, thrombosis, breakage, migration, displacement) including rare but life-threatening complications such as pericardial effusion and cardiac tamponade.

Incidence of pericardial effusion/cardiac tamponade has been previously reported in literature between 0.07% and 2% of PICLs placement [1-8]. Pericardial effusion may be more likely when the catheter tip is placed within the heart outline on X-ray examination; however, positioning the tip outside the heart does not completely prevent it.

As timely diagnosis and pericaridocentesis has been proven to be life-saving, this complication should be considered in any newborn with an inserted central line who presents with cardiorespiratory instability.

We report the case of a successful pericardiocentesis performed in a ELBW neonate who presented with cardiac tamponade 18 days after the placement of a PICL.

## Case presentation

A 620 g Caucasian female, was delivered at our Hospital after 25 weeks of gestation by caesarean section secondary to maternal congestive heart failure from dilated cardiomiopathy. Apgars were 9 and 9 at 1 and 5 minutes, respectively. She was intubated at birth and received surfactant. A 27 G polyurethane (PremiCath^P^) central venous catheter was inserted through the right basilic vein on day 1. Chest X-ray after insertion confirmed optimal positioning, the catheter tip being at the junction of inferior vena cava and right atrium. The neonate was mechanically ventilated for 14 days and then started nasal continuous positive airway pressure (CPAP) ventilation.

On day 18 the baby weight was 670 g. She was still on CPAP with a FiO_2 _of 0.4 and a PEEP of 4 cmH_2_O. Arterial blood gas analysis showed good parameters (pH: 7.3, pCO_2_: 56 mmHg, PO_2 _39 mmHg), oxygen saturation values were stable (97%). The neonate was on partial parenteral nutrition, receiving 50 ml of formula milk and 36 ml of parenteral nutrition administered via the peripheral venous route. Unexpectedly, at 10:30 p.m. the infant started presenting worse saturations (92-94%) and her oxygen requirement increased form 0.4 to 0.6. At 12:00 p.m. she suddenly became pale, presented repeated and prolonged desaturations and needed to be ventilated. Despite an improvement in oxygen saturation immediately after positive-pressure ventilation (PPV) with bag and mask, the infant's clinical conditions worsened again and she needed to be reintubated. Chest X-ray showed the catheter tip at the connection of the inferior vena cava with the right atrium and no lung opacities. Regardless of synchronized intermittent mandatory ventilation (SIMV) (PIP 18, PEEP 3, FiO_2 _65%, rate 38, Ti 0.35), at 1.00 a.m. there was a sudden deterioration with cardiorespiratory instability: the oxygen requirement increased up to 0.9 and the baby developed bradycardia (70 bpm). Brachial arterial pressure was not detectable. Lung auscultation revealed coarse crakles, while cardiac auscultation revealed "distant" heart sounds. Dopamine (5 μg/kg per min) and furosemide (1.2 mg "una tantum") were administered.

The transthoracic echocardiography demonstrated marked pericardial effusion with evidence of cardiac tamponade (additional file 1). Urgent periocardiocentesis was planned; in the meanwhile cardiac arrest ensued (additional file 2). Subxiphoid pericardiocentesis was immediately performed using a 22-gauge needle. Totally 2 ml of milky fluid was collected (see additional file 3). Skin appearance of the neonate improved rapidly after the procedure, her vital signs immediately returned to normal and her oxygen requirement instantly decreased from 0.9 to 0.5 and after a few minutes to 0.35. Echocardiography ten minutes later showed increased contractility with partial resolution of the pericardial effusion (additional file 4). At 2.00 a.m. the PICL was pulled back into the subclavian vein and inotropic support could be discontinued; two hours later ventilatory parameters could be reset (PIP 20, PEEP 3, FiO_2 _40%, rate 40).

The morning after the neonate's clinical conditions were stable and arterial blood gas analysis showed good parameters (pH: 7.4, pCO_2_: 50 mmHg, PO_2 _46 mmHg). Biochemical analysis of the obtained pericardial fluid showed it to be consistent with the composition of the hyperosmolar TPN solution the neonate was receiving through the catheter. Ecocardiography showed normal cardiac filling and contractility (additional file 5).

The subsequent clinical course of the baby was uneventful. One year follow up echocardiograms showed no re-accumulation of the fluid and normal cardiac filling and contractility.

## Conclusion

In the case of this ELBW neonate with PICL who presented with cardiac tamponade, timely diagnosis and pericaridocentesis using echocardiography resulted successful. Our experience shows that basic echocardiography is an easily accessible bedside assessment tool that may be life-saving and that subxiphoid percutaneous approach for the dreinage of pericardial effusion is safe, effective and easily performed even in a ELBW neonate.

As an emergency treatment may be life saving, cardiac tamponade must be kept in mind as a cause of sudden deterioration of very low birth weight neonates and promptly diagnosed. In this case report, the complication occurred 18 days after the placement of the central line and in spite of a presumed satisfactory position of the catheter tip. Thus, cardiac tamponade should be considered in any newborn with a peripherally inserted central catheter who presents with sudden deterioration, even when lines are believed to be placed correctly.

## Consent

Written informed consent was obtained from the parents of the patient for publication of this case reports and accompanying images and videos. A copy of written consent is available for review by the journal's Editor-in-Chief.

## Competing interests

The authors declare that they have no competing interests.

## Authors' contributions

All authors contributed to acquisition of case details and the analysis and interpretation of them. EP wrote the first draft of the manuscript, AP and MF revised the manuscript.

All the authors have given final approval of this version to be published.

## Supplementary Material

Additional file 1 **Echocardiogram, 1:53 a.m. **2D-Echocardiogram, subcostal view: massive pericardial effusion. Click here for file

Additional file 2 **Echocardiogram, 1:56 a.m. **2D-Echocardiogram, subcostal view. Swinging heart with collapse of right cavities and severely depressed contractility Click here for file

Additional file 3 **Echocardiogram, 1:59 a.m. **2D-Echocardiogram, subcostal view, during pericardiocentesis. Reduced pericardial effusion and improvement of right heart filling and contractility. Click here for file

Additional file 4 **Echocardiogram, 2:10 a.m. **2D-Echocardiogram, subcostal view. Further improvement of filling and contractility Click here for file

Additional file 5 **Echocardiogram, 8:28 a.m. **2D-Echocardiogram, subcostal view. Mild pericardial effusion with normal filling and contractility. Click here for file
